# Different Susceptibility of T and B Cells to Cladribine Depends On Their Levels of Deoxycytidine Kinase Activity Linked to Activation Status

**DOI:** 10.1007/s11481-021-09994-3

**Published:** 2021-04-14

**Authors:** Federico Carlini, Federico Ivaldi, Francesca Gualandi, Ursula Boschert, Diego Centonze, Giuseppe Matarese, Marco Salvetti, Nicole Kerlero de Rosbo, Antonio Uccelli

**Affiliations:** 1 Department of Neurosciences, Rehabilitation, Ophthalmology, Genetics, Maternal and Child Health (F.C, A.U.), F.I., N.KdeR., A.U.) and Centre of Excellence for Biomedical Research (F.C., A.U.), University of Genova, Genoa, Italy; 2 IRCCS Ospedale Policlinico San Martino (F.G., A.U.), Genoa, Italy; 3 Synaptic Immunopathology Lab (D.C.), Department of Systems Medicine, Tor Vergata University, Rome, Italy; 4 IRCCS Neuromed, Unit of Neurology (D.C.), IS Pozzilli, Italy; 5 Treg Cell Lab (G.M.), Dipartimento di Medicina Molecolare e Biotecnologie Mediche, Università degli Studi di Napoli Federico II, Naples, Italy; 6 Laboratorio di Immunologia, Istituto di Endocrinologia e Oncologia Sperimentale (G.M.), Consiglio Nazionale delle Ricerche (IEOS- CNR), Naples, Italy; 7 Department of Neuroscience, Mental Health and Sensory Organs (M.S.), Faculty of Medicine and Psychology, Centre for Experimental Neurological Therapies, S. Andrea Hospital, Sapienza University, Rome, Italy; 8 IRCCS Istituto Neurologico Mediterraneo (INM) Neuromed (M.S.), Rome, Italy; 9 Ares Trading SA, Switzerland, an affiliate of Merck KGaA, (U.B.), Darmstadt, Germany

**Keywords:** Multiple sclerosis, T cells, B cells, Deoxycytidine kinase, 5’ deoxynucleotidase, Cladribine

## Abstract

**Abstract:**

Deoxycytidine kinase (dCK) and 5’ deoxynucleotidase (NT5C2) are involved in metabolism of cladribine (2CdA), the immunomodulatory drug for multiple sclerosis; by mediating phosphorylation (activation) or phosphorolysis (deactivation) of 2CdA, respectively, these enzymes promote or prevent its accumulation in the cell, which leads to cell death. In particular, lymphocytes which present with a high intracellular dCK/NT5C2 ratio are more sensitive to 2CdA than other immune cells. We aim at determining if the expression of these enzymes and/or their activity differ in specific progenitor and mature immune cells and are influenced by cellular activation and/or exposure to 2CdA. Flow cytometry analysis showed no difference in dCK/NT5C2 ratio in progenitor and mature immune cells. 2CdA induced apoptosis in stimulated T and B cells and unstimulated B cells. dCK expression was enhanced by 2CdA at mRNA and protein levels in activated T cells and mRNA level in activated B cells. dCK activity, measured through an in-house luminescence release enzyme assay was higher in activated T and B cells, and such an increase was abrogated in activated B cells, but not T cells, upon exposure to 2CdA. These results reveal an important relationship between dCK activity and the effect of 2CdA on B and T cells, according to their activation status. Further study is warranted to evaluate whether dCK activity could, in the future, be a suitable predictive biomarker of lymphocyte response to 2CdA.

**Graphical Abstract:**

**Supplementary Information:**

The online version contains supplementary material available at 10.1007/s11481-021-09994-3.

## Introduction

Cladribine (2CdA), an adenosine antimetabolite preferentially targeting lymphocytes, was recently approved in the European Union as oral treatment for highly active relapsing multiple sclerosis (Comi et al. 2019), the most common demyelinating central nervous system disorder, in which T and B cells play a crucial role (Compston and Coles 2008; Peterson et al. 2001; Wekerle 2017). 2CdA has a chlorine substitution for hydrogen in its purine ring, which renders it resistant to adenosine deaminase (Beutler 1992), an enzyme regulating both extracellular and intracellular levels of nucleotides (Cekic and Linden 2016; Dong et al. 2016; Yegutkin 2008). 2CdA undergoes sequential phosphorylation, the first step of which is catalysed by deoxycytidine kinase (dCK), to generate the active mononucleotide 2-chlorodeoxyadenosine 5’-triphosphate (2CdATP), which accumulates in the cytoplasm and incorporates into DNA, thereby blocking its synthesis (Leist and Weissert 2011; Sasvari-Szekely et al. 1994) and leading to cell death. Phosphorolysis of 2CdATP by 5’-nucleotidases reverts its activation and thereby, its accumulation into the cell. 5’-nucleotidase cytosolic type 2 (NT5C2) is the most represented in all immune cell subtypes (Salvat et al. 2009), and the preferential action of 2CdA on lymphocytes is related to their high intracellular dCK/NT5C2 ratio, which results in accumulation of 2CdATP (Leist and Weissert 2011). dCK expression and activity are enhanced in activated lymphocytes suggesting higher sensitivity to 2CdA (Staub 2006; Toy et al. 2010). Reported differences in T- and B-cell reconstitution (Comi et al. 2019; Stuve et al. 2019) might therefore be explained by a distinct sensitivity of lymphoid subsets and/or their progenitors to 2CdA, due to differences in dCK expression and/or activity according to their activation status.

Our objective was to determine whether or not the expression and/or activity of the enzymes (dCK and NT5C2) involved in 2CdA metabolism differ in selected progenitor and mature immune cell subsets ex vivo, or upon activation of mature T and B cells exposed to 2CdA in vitro.

## Methods

### Cell Collection and Isolation

Bone marrow (BM) samples were collected by aspiration from the posterior iliac crests of healthy donors undergoing stem cell harvesting, at the Unità Operativa Ematologia, Ospedale Policlinico San Martino -IRCCS, Genova, Italy. Mononuclear cells were isolated from BM samples by Ficoll-gradient centrifugation (Bignold and Ferrante 1987), cryopreserved, and stored under liquid N_2_.

Peripheral blood mononuclear cells (PBMC) were isolated from whole blood of healthy donors by Ficoll-gradient centrifugation.

### Flow Cytometry

Cryopreserved BM mononuclear cells were thawed and potential dead cells removed through Ficoll-gradient centrifugation. Common lymphoid progenitors (CLP) and common myeloid progenitors (CMP) were identified as Lineage (Lin)- CD34 + CD38 + CD10 + and Lin- CD34 + CD38 + CD123^Low^ CD45RA-, respectively, using fluorescent labelled monoclonal antibodies (mAb) (Table 1). Immune cell subsets relevant to MS pathogenesis were assessed in PBMCs as previously described (Cellerino et al. 2020), using Lyotubes (BD Biosciences, Italy, Cat. No. 625,148) optimised to monitor broad subpopulations of effector CD4 + T cells (Th1, CD3 + CD4 + CXCR3 + CCR6-CD161-; Th17, CD3 + CD4 + CXCR3-CCR6 + CD161 + CCR4+; Th1/17, CD3 + CD4 + CCR6 + CD161 + CXCR3hiCCR4low), regulatory CD3 + CD4 + T cells (Total Treg, CD25 + CD127-; T naïve CD45RA + CD25low), regulatory CD3 + CD8 + T cells (Treg, CD28 Treg CD28- CD127-), effector B cells (B memory CD19 + CD14-CD24highCD38-; B mature CD19 + CD14-CD24lowCD38low), regulatory B cells (CD19 + CD14-CD24highCD38high), effector NK cells (CD3-CD16 + CD56dim), and regulatory NK cells (CD3-CD16 + CD56bright) (Cellerino et al. 2020). Progenitor cells and mature cell subsets were first stained for surface markers; they were then permeabilized with Cytofix/Cytoperm™ kit (BD Bioscence, Italy) and stained for the intracellular enzymes, using the primary mouse mAb anti-dCK (clone OTI3F5, MA5-25500 Thermo Fisher Scientific, Italy) and rabbit anti-NT5C2 polyclonal (p)Ab (ab96084, Abcam, UK) antibodies, followed by secondary Alexa Fluor® 488 anti-mouse IgG2a (Biolegend, CA) and Alexa Fluor® 488 anti-rabbit IgG (Biolegend, CA) antibodies. Fixable Viability Stain (FVS)-780 was used to exclude dead cells from the analysis. The gating strategies for CLP/CMP and the immune cells subsets are shown in Supplementary Figures 1 and 2.

**Table 1:** Antibody and fluorochrome panels to identify CLP and CMP from bone-marrow mononuclear cells

	Fluorochrome					
	PB *	PerCP*	PE*	APC*	AmCyan *	PE-Cy7*
Antibody specificity	Lineage Cocktail (CD3, CD14, CD16, CD19, CD20, CD56)	CD10	CD34	CD38	CD45RA	CD123
Clone	SK7; 3G8; MφP9; SJ25C1; L27; NCAM16.2	HI10a	8G12	HB-7	HI100	9F5
CLP	−	+	+	+	/	/
CMP	−	/	+	+	−	low

+ and - signs mean positivity and negativity for a specific marker used for the gating strategy of a specific cell subset; / indicates a marker that is not used for the gating strategy

*PB Pacific blue, *PerCP* Peridinin Chlorophyll Protein Complex, *PE* phycoerythrin, *APC* Allophycocyanin, *PE-Cy7* Peridinin chlorophyll protein-Cy5.5

### In-vitro CD4 + T- and CD19 + B-cell Activation: Assessment of Cell Viability And/or Proliferation

CD4 + T cells and CD19 + B cells were isolated by negative selection from freshly isolated PBMC using Human CD4 + T- and CD19 + B-cell Isolation Kits (Milteny, Germany) according to manufacturer’s instructions. The purity of CD4 + T cells and CD19 + B cells after isolation was 97.8 (SD = 1.8) and 98.5 % (SD = 2.1), respectively, as verified by flow cytometry using anti-CD4 + mAb (clone SK7, V500-C, BD Bioscence, Italy) and anti-CD19 + mAb (SJ25CL, PE-Cy7, BD Bioscence, Italy).

Freshly isolated CD4 + T cells or CD19 + B cells were seeded in 24-well flat-bottom plates (500,000 cells/well) and activated with Dynabeads™ Human T-Activator CD3/CD28 kit (25 µl/10^6^ cells; Thermo Fisher Scientific, Italy) or with 10 ng/ml IL-15 (VWR, Italy) plus 5 µM CpG (InvivoGen, France) (Gupta et al. 2018), respectively, for 48 and 72 h in RPMI medium containing 10 % foetal bovine serum and 1 % Penicillin-Streptomycin (Thermo Fisher Scientific, Italy). 2CdA was added (Merck KGaA, Germany) at different concentrations (see Results) at the same time as the T- or B-cell activators, for the duration of the culture. Viability of CD4 + T cells and CD19 + B cells was assessed by flow cytometry with Annexin V (Biolegend, CA) and propidium iodide (Merck KGaA, Germany).

### Real‐time PCR

RNA extracted from CD4 + T cells and CD19 + B cells using QIAzol Lysis Reagent (Qiagen, France) was reverse-transcribed into cDNA using QuantiTect Reverse Transcription Kit (Qiagen, France) according to manufacturer’s protocol. Real-time PCR was performed using LightCycler 480 (Roche Applied Science, Germany) in a final reaction volume of 20 µl containing 10 ng cDNA, 2 µl of primers/probe mix (0.5 µM and 0.02 µM final concentration for primers and probes, respectively), and 10 µl FastStart Essential DNA Probes Master ready-to-use reaction mix (Roche Applied Science, Germany). Measurement of glyceraldehyde 3-phosphate dehydrogenase (*GAPDH*) mRNA was used for normalization of expression data. Primers and probe for DCK were designed from the mRNA reference sequence (NM_000788.2) [DCK : Forward primer (Fw) 5’-CCACCCCGCCCAAGAGA-3’; Reverse primer (Rw) 5’-CTTCCCTGCAGCGATGTTCCC-3’; Probe (Pb) FAM-TGCCCGTCTTTCTCAGCCAGCTCT-BBQ]. Since both *NT5C2* and *GAPDH* possess multiple mRNA isoforms, sequences of primers and probes were designed to target all the different transcript variants listed in the National Center for Biotechnology Information database (Sayers et al. 2020) [*NT5C2* : Fw 5’-GGCAAGCTGAAAATTGGTACCT-3’; Rw 5’-TCGTATCAGAAGAACCTCCTGAGTAG-3’; Pb FAM-ACAGGGCCCCTACAGCATGGTATCG-BBQ; GAPDH: Fw 5’-TCACCACCATGGAGAAGGC-3’; Rw 5’-GCTAAGCAGTTGGTGGTGCA-3’; Pb FAM-ATGCCCCCATGTTCGTCATGGGTGT-BBQ]. Quantification was carried out using the relative standard curve method as previously described (Carlini et al. 2017). An efficiency equal to two was assumed (Bustin et al. 2009).

### Western Blotting

CD4 + T cells were lysed using RIPA buffer containing protease inhibitors (Roche Applied Science, Germany). Protein sample load was based on the number of cells (3 × 10^5^ cells per lane) to quantify the cellular changes in expression of the protein tested, under different experimental conditions. Electrophoresis was performed on a 4–15 % Mini-PROTEAN® TGX™ Precast Gels (BioRad, CA), using Mini-PROTEAN® Tetra Vertical Electrophoresis Cell (BioRad, CA) and transferred to nitrocellulose membrane (BioRad, CA) using XCell II™ Blot Module (Thermo Fisher Scientific, Italy). Membranes were blocked for 2 h with 5 % BSA in PBS/0.1 % Tween 20 and incubated overnight with primary antibodies, mAb anti-dCK (clone OTI3F5, 1:10,000; Thermo Fisher Scientific, Italy), pAb NT5C2 (ab96084, 1:5000; Abcam, UK), mAb anti-vinculin (VCN) (clone hVIN-1, 1:20,000; Merck KGaA, Germany). Membranes were washed with PBS/Tween 20 and incubated for 1 h with secondary horseradish peroxidase-conjugated antibodies (anti-mouse IgG 1:10,000, NA931; anti-rabbit IgG, 1:10,000, AP307P; Merck KGaA, Germany). Reactive bands were visualized using ECL Plus (Thermo Fisher Scientific, Italy). Densitometric analysis for relative protein quantification was performed with ImageJ software (NIH) and normalized to loading control protein, VCN.

### Assessment of dCK Activity

**Fig. 1:**
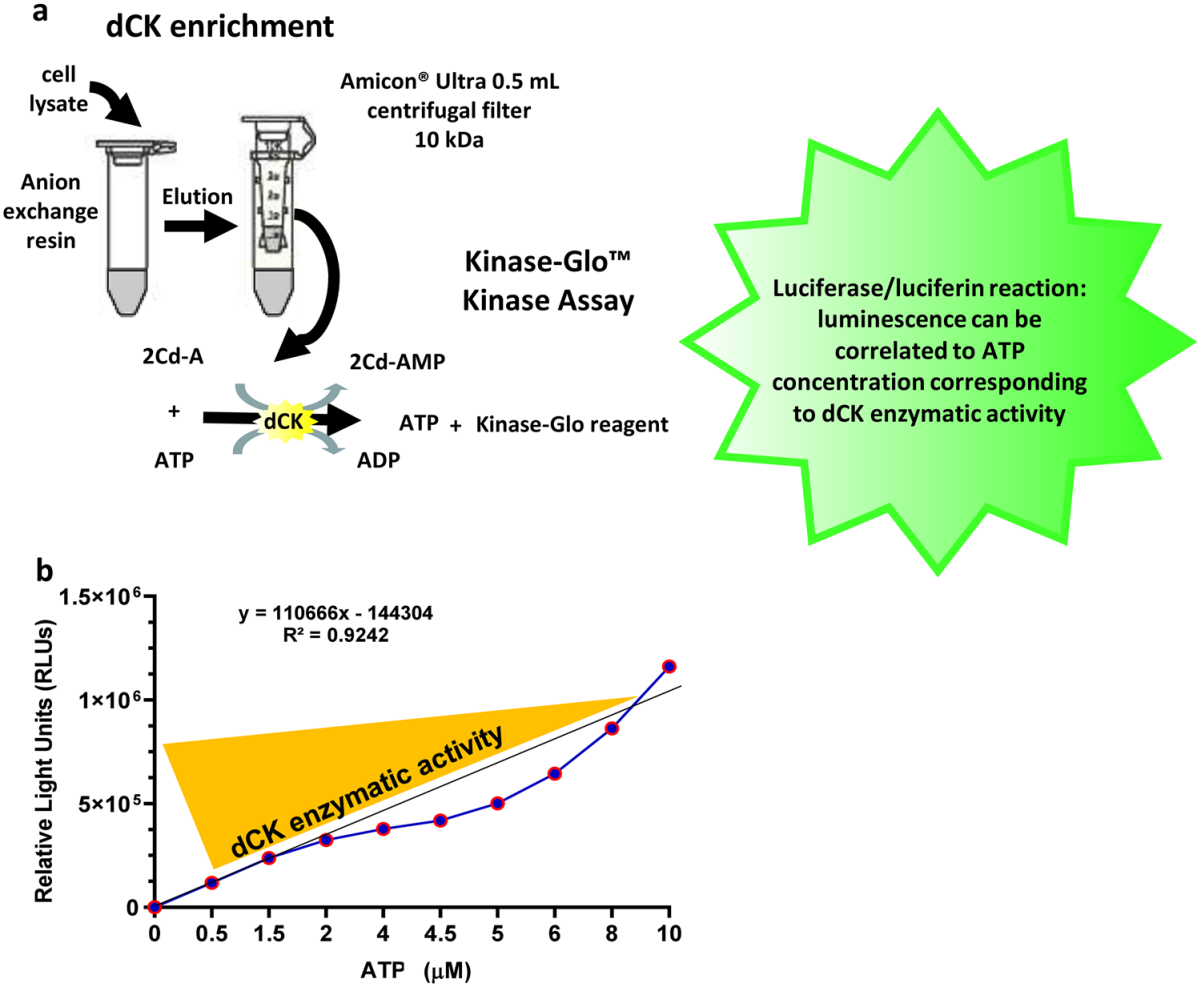
Schematic representation of dCK enrichment procedure and assay of dCK activity. (**a**) The procedure involves the use of Q Sepharose ® anionic exchange beads to enrich dCK from cell lysates followed by concentration of the eluted fraction using Amicon® Ultra 0.5 ml Centrifugal Filters. Assessment of dCK activity is performed using the Kinase Glo ™ kit which measures the kinase activity indirectly by converting ATP left in the reaction mixture to light (luminescence) through luciferin/luciferase-dependent reaction. (**b**) Standard curve reflecting luciferase conversion of ATP. Different amount (µM) of ATP are converted into a sustained light output (λ = 570 nm), expressed as relative light units (RLUs), through a luciferin/luciferase-dependent reaction. dCK activity is inversely proportional to residual ATP concentration in the reaction medium

Cells (5 × 10^6^ collected from 10 wells of 24-well plates) were lysed using CytoBuster™ Protein Extraction Reagent (Merck KGaA, Germany) following manufacturer’s protocol. Enrichment of dCK from cell lysates was performed essentially as described (Hao et al. 2014) with some modifications. Briefly, 0.5 ml cell lysate were incubated in a 1.5 ml vial for 5 min with Q Sepharose ® anionic exchange beads (1:3 volume of lysate; Merck KGaA, Germany) pre-equilibrated with 0.01 M Tris-HCl buffer (pH 7.0). The beads were washed three times with 0.01 M Tris-HCl buffer (pH 7.0) containing 5 mM β-mercaptoethanol. dCK elution was repeated three times using the same Tris-HCl buffer containing 0.5 M NaCl (1:1 initial volume of lysate). dCK-enriched extracts were concentrated on Amicon® Ultra 0.5 ml Centrifugal Filter devices (Merck KGaA, Germany) with 10-kDa cut-off. The whole procedure was conducted at 4° C (Fig. 1a).

Quantification of the enzymatic activity was conducted on dCK-enriched extracts using Kinase-Glo ™ reagents (Promega, Wisconsin, USA) according to manufacturer’s protocol. Briefly, dCK-enriched extracts (1 µg) in Kinase Reaction Buffer (40 mM Tris pH 7.5, 20 mM MgCl_2_, 0.1 mg/ml bovine serum albumin) were incubated in 0.5 ml Eppendorf tubes at 37 °C in the presence of 10 µM ATP and 30 µM 2CdA (final volume 5 µl) for 0, 5, and 10 min. The reaction mixtures were moved to White Bio-One 384-well plates (Greiner Bio-One, Italy) and incubated for 10 min at room temperature in the presence of 10 µl Kinase-Glo® Reagent. The luminescence emitted by the samples was read on a Spark® Multimode Microplate Reader (Tecan, Switzerland). dCK activity was calculated as luminescence/min after subtracting the blank sample value (Kinase Reaction Buffer alone) (Fig. 1b). dCK-enriched extract without 2CdA was used to control that the activity observed was not due to other kinase contaminants potentially present after dCK enrichment procedure.

## Statistics

Statistical analyses were performed using GraphPad Prism 8.0 (GraphPad Software, La Jolla California USA, www.graphpad.com). Normality distribution for numerical data was tested through Kolmogorov-Smirnov and Shapiro–Wilk tests. Statistical associations were tested using nonparametric statistics. For two modalities, the Kolmogorov-Smirnov test was used. Kruskal-Wallis one-way ANOVA followed by Dunn post-hoc test was used for more than two modalities. *P* value ≤ 0.05 was considered as significant.

## Results

### Flow Cytometry Analysis Shows No Difference in dCK/NT5C2 Ratio in Immune Cell Progenitors and Mature Lymphocyte Subsets

**Fig. 2:**
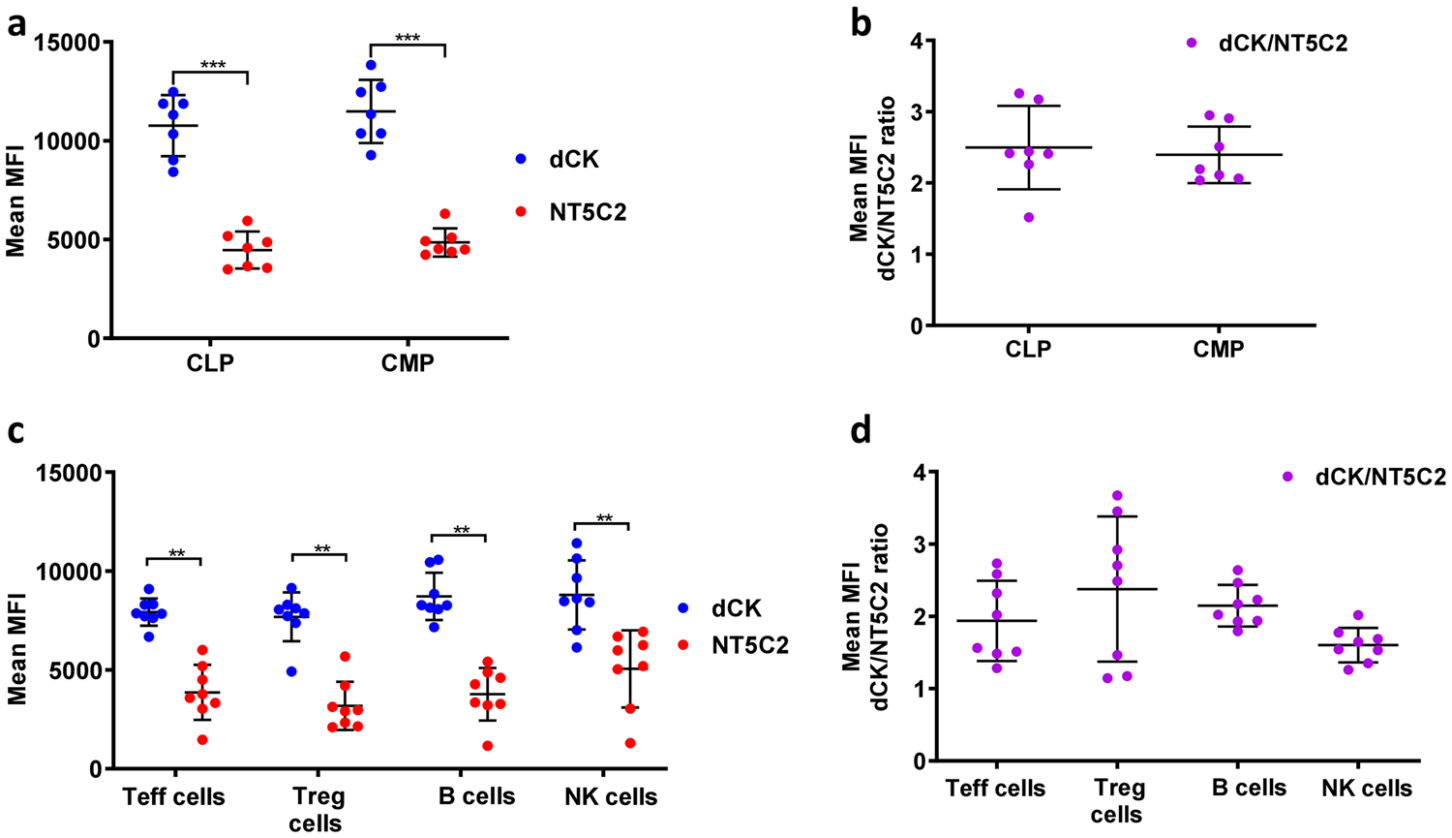
dCK and NT5C2 expression/ratio do not differ between progenitor or mature immune cell populations. (**a**) Flow cytometry quantification of dCK and NT5C2 protein expression, measured as mean fluorescence intensity (MFI), and (**b**) MFI dCK/NT5C2 ratio in CLP and CMP populations in bone-marrow cells. (**c**) Flow cytometry quantification of dCK and NT5C2 protein expression, measured as MFI, and (**d**) MFI dCK/NT5C2 ratio in Teff, Treg, B, and NK cells in PBMC. MFI values for dCK and NT5C2 were standardized according to “isotype” and “unstained”, respectively. Data are presented for bone-marrow cells and PBMC isolated from 7 and 8 healthy donors, respectively, assayed in independent experiments; ** *P* < 0.01,*** *P* < 0.001

**Fig. 3:**
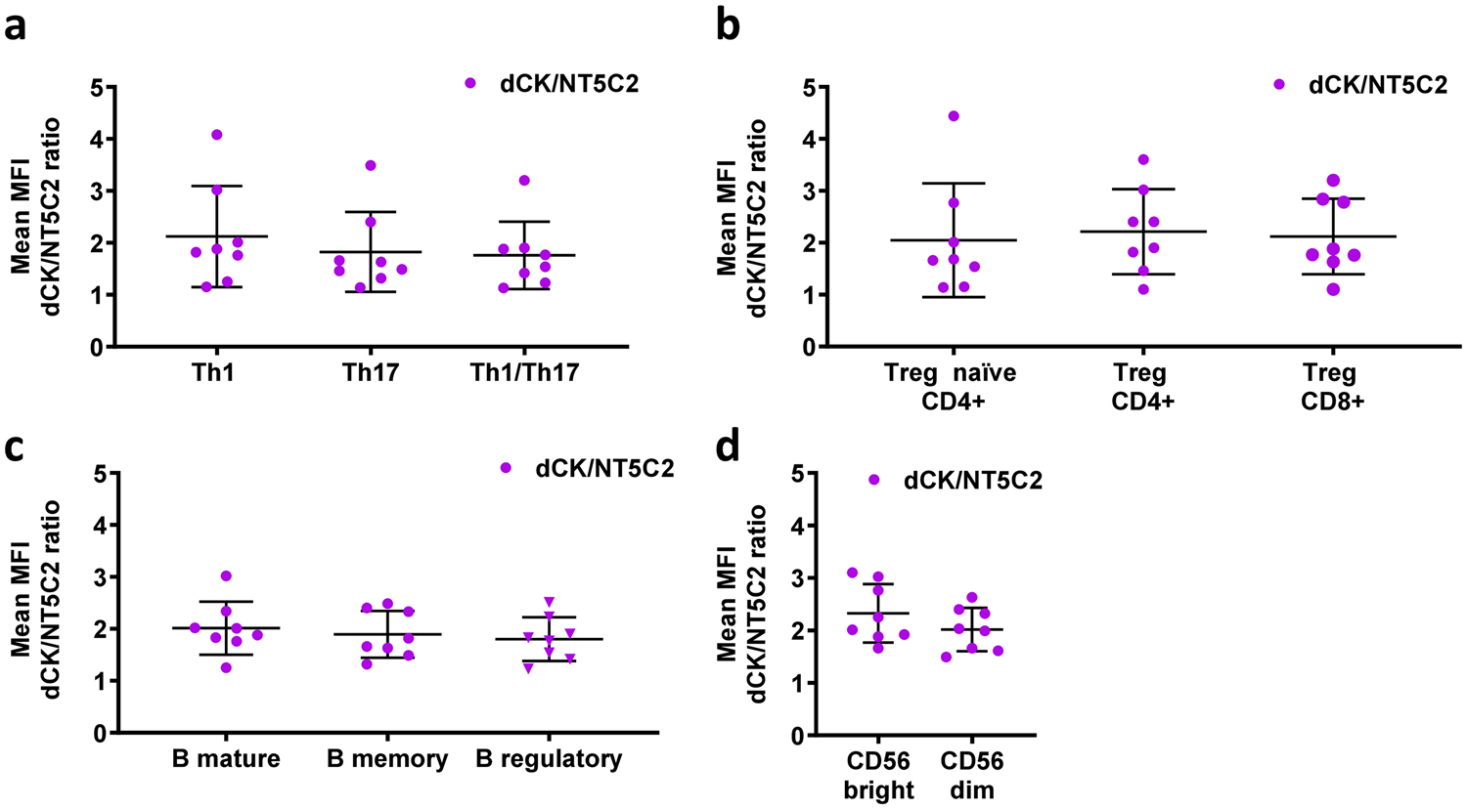
The ratios of dCK and NT5C2 expression (MFI) do not differ between subsets within the T, B, and NK cell populations. Quantification of the MFI dCK/NT5C2 ratio from flow cytometry analysis of Teff-cell (**a**), Treg-cell (**b**), B-cell (**c**), and NK-cell (**d**) subsets, in PBMC. Data are presented for PBMC isolated from 8 healthy donors assayed in independent experiments

Protein expression of the enzymes dCK and NT5C2 in common lymphoid (CLP) and common myeloid progenitors (CMP) was assessed by flow cytometry in bone marrow from eight healthy individuals. As can be seen in Fig. 2, the expression of the enzymes was similar in both cell subsets, with dCK expression being higher than that of NT5C2 (Fig. 2a); accordingly, no significant difference was observed in dCK/NT5C2 ratio between CLP and CMP cells (Fig. 2b). Similarly, the protein expression of dCK was higher than that of NT5C2 in subpopulations of mature immune cells, effector (Teff) and regulatory (Treg) T cells, B cells, and natural killer (NK) cells, in isolated PBMC, but it did not differ between cell types (Fig. 2c). This latter finding is in line with data obtained at mRNA level available from a public database (http://www.biogps.org, (Ceronie et al. 2018; Leist and Weissert 2011). We did not observe any difference in the dCK/NT5C2 ratio between B cells and other immune cell types (Fig. 2d), despite the reported greater sensitivity of B cells to 2CdA (Stuve et al. 2019), nor were there differences in this ratio between sub-subsets of Teff, Treg, B, and NK cells that are relevant to MS (Fig. 3).

### 2CdA Induces Apoptosis in Stimulated T and B Cells and in Unstimulated B Cells

**Fig. 4:**
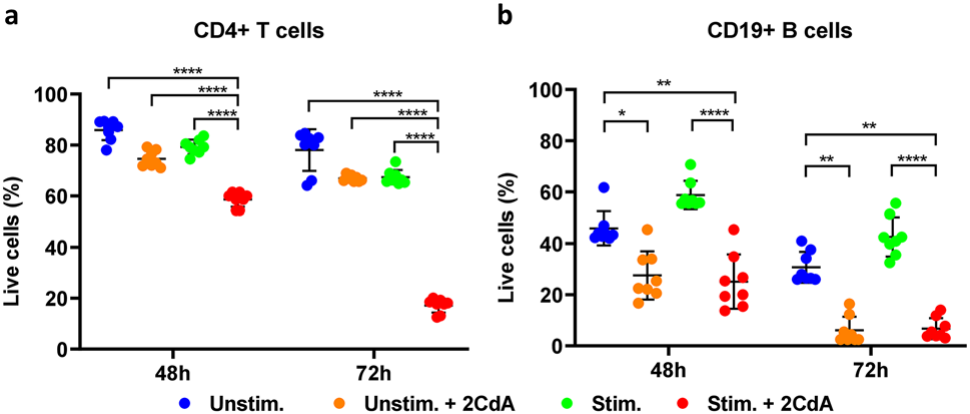
2CdA treatment reduces T- and B-cell viability. Flow cytometry analysis of viability of (**a**) CD4 + T cells and (b) CD19 + B cells treated as indicated for 48 and 72 h, using Annexin V/propidium iodide labeling of apoptotic cells and necrotic cells, respectively. Data are presented for cells from 8 (CD4 + T cells) or 5 (CD19 + B cells) healthy donors assayed in independent experiments; * *P* < 0.05, ** *P* < 0.01, **** *P* < 0.0001. *Unstim.* = unstimulated cells; *Unstim. + 2CdA* = unstimulated cells treated with 2CdA (500 nM); *Stim.* = cells stimulated with anti-CD3/CD28 beads (CD4 + T cells) or IL-15/CpG (CD19 + B cells); *Stim. + 2CdA* = cells (CD4 + T cells or CD19 + B cells) stimulated as before and treated with 2CdA (500 nM)

We have postulated that 2CdA preferentially acts on activated/proliferating cells. Accordingly, we assessed the effect of 2CdA on the viability and function of CD4 + T cells stimulated by T-cell receptor triggering with anti-CD3/CD28 antibodies. We tested a range of different concentrations of 2CdA (10 nM-1 µM) on CD4 + T cells as previously described (Korsen et al. 2015), and 500 nM was the optimal 2CdA concentration to observe an impact on viability (data not shown); we have used this concentration of 2CdA in the subsequent experiments of this study. Indeed, such a concentration was also on par with the in-vivo concentration of 2CdA reported in the blood of multiple sclerosis patients treated with 10 mg cladribine (Mavenclad®) tablets (Hermann et al. 2019). Exposure of activated CD4 + T cells to 2CdA for 48 h resulted in a 26.5 % reduction in live cells (Fig. 4a). After 72 h, the presence of 2CdA in the culture further reduced the live cell population to 74.2 % of the original cell numbers (Fig. 4a). No significant 2CdA effect was observed in unstimulated CD4 + T cells in the presence or absence of 2CdA (Fig. 4a). In contrast to what was observed with CD4 + T cells, exposure to 2CdA reduced viability of B cells unstimulated and stimulated with IL-15/CpG, by 39.6 and 65.08 %, respectively, at 48 h and by 74.72 and 82.32 %, respectively, at 72 h (Fig. 4b), as compared to B cells not exposed to 2CdA (Fig. 4b).

### 2CdA Treatment is Associated With an Upregulated Expression of *DCK* and *NT5C2* mRNA in T and B Cells and of dCK Protein in T Cells

**Fig. 5:**
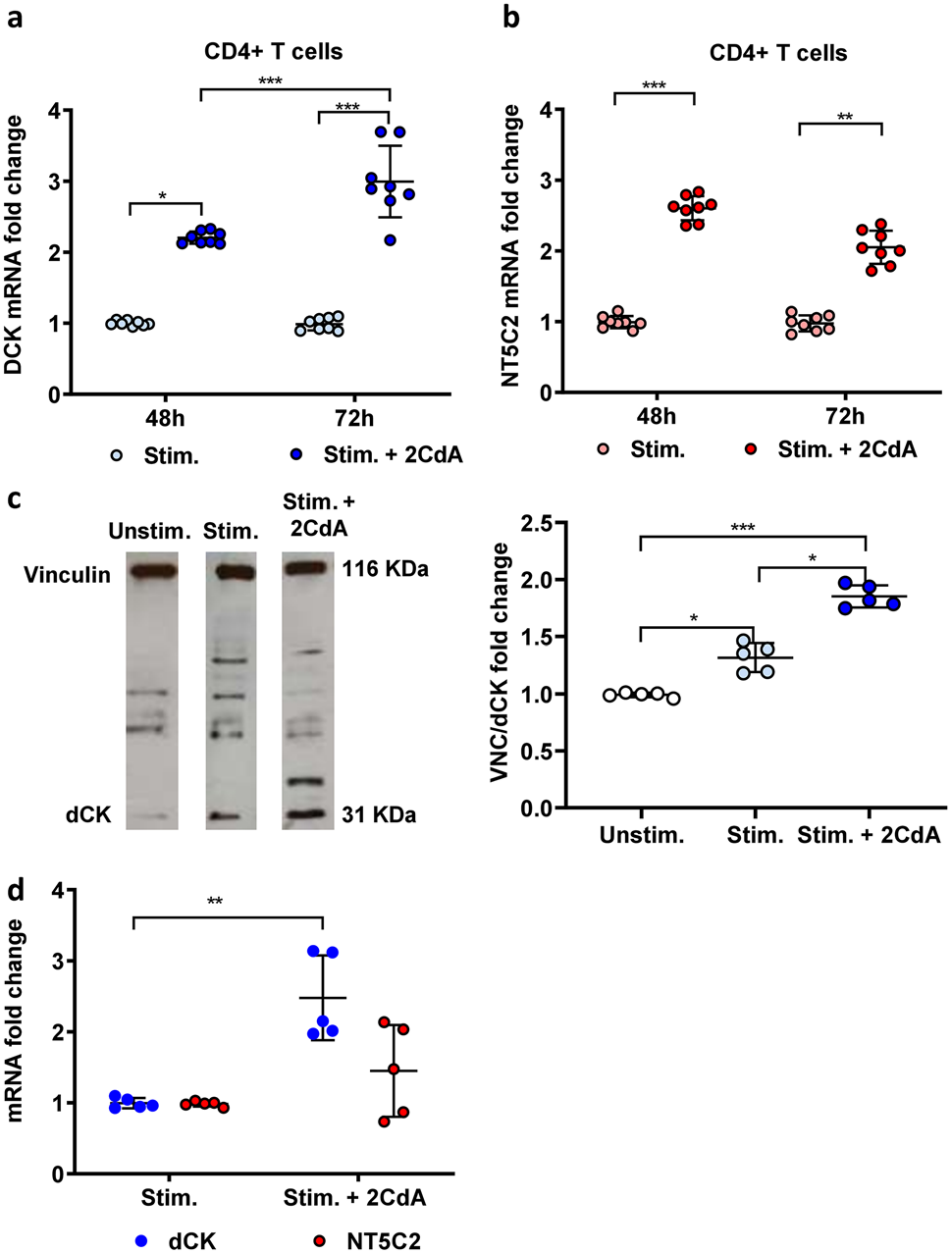
***DCK*** and ***NT5C2*** mRNA and/or protein expression are upregulated by 2CdA in T and B cells. (**a**) *DCK* and (**b**) *NT5C2* mRNA expression measured by Real-time PCR in CD4 + T cells stimulated with anti-CD3/CD28 beads for 48 and 72 h in the presence or absence of 2CdA (500 nM). The mean ΔCT + SD baseline levels of expression of *DCK* and *NT5C2* mRNA in unstimulated cells were 9.98 + 0.41 and 8.5 + 0.13, respectively. Data are presented for T cells isolated from 8 healthy donors assayed in independent experiments; * *P* < 0.05, ** *P* < 0.01, *** *P* < 0.001. (**c**) Representative Western blot (left panel) and quantification (right panel) of dCK in stimulated CD4 + T cells exposed or not to 2CdA (500 nM) for 72 h. Data were normalized to loading control protein, VCN. Fold changes were calculated using Unstim. controls as reference. Data are presented for T cells isolated from 5 healthy donors assayed in independent experiments; * *P* < 0.05, ** *P* < 0.01. Unstim. = unstimulated cells;. Stim. = CD4 + T cells stimulated with anti-CD3/CD28 beads; Stim. + 2CdA = CD4 + T cells stimulated with anti-CD3/CD28 beads and treated with 2CdA (500 nM). (**d**) *DCK* and *NT5C2* mRNA expression measured by Real-time PCR in CD19 + B cells stimulated anti-IL-15/CpG for 72 h in the presence or absence of 2CdA. The mean ΔCT + SD baseline levels of expression of *DCK* and *NT5C2* mRNA in unstimulated cells were 10.5 + 0.40 and 7.475 + 0.12, respectively. Data are presented for B cells isolated from 5 healthy donors assayed in independent experiments; * *P* < 0.05. Stim. = CD19 + B cells activated with IL-15/CpG; Stim. + 2CdA = CD19 + B cells stimulated with IL-15/CpG treated with 2CdA (500 nM). *GAPDH* mRNA was used as endogenous control to normalize the expression data in the PCR analyses

Upon stimulation, CD4 + T cells exposed to 2CdA for 48 h showed an upregulation in both *DCK* and *NT5C2* mRNA expression as compared to stimulated CD4 + T cells not exposed to 2CdA (Fig. 5a and b). After 72 h, there was a further increase in *DCK* mRNA expression in the cells treated with 2CdA (Fig. 5a). Upregulation of dCK expression was confirmed at protein level in stimulated CD4 + T cells exposed to 2CdA after 72 h (Fig. 5c). CD4 + T-cell stimulation resulted in a significant upregulation of dCK protein expression compared to unstimulated cells (Fig. 5c). In contrast, there was no significant difference in *DCK* and *NT5C2* mRNA expression between unstimulated and stimulated B cells (data not shown). As observed with activated CD4 + T cells exposed to 2CdA, *DCK* mRNA expression was increased in IL-15/CpG-stimulated B cells in the presence, but not the absence, of 2CdA (Fig. 5d). The expression of *NT5C2* was not affected by exposure to 2CdA (Fig. 5d).

### dCK Activity Augments in Activated CD4 + T Cells and CD19 + B Cells, an Increase Abrogated in Activated B Cells Upon Exposure to 2CdA

Although the expression of dCK at mRNA and protein levels could be partially predictive of potential susceptibility to 2CdA in specific immune cell subsets, the enzyme activity most likely plays a dominant role for the differential accumulation of 2CdATP within the cells. Thus, only measuring dCK at expression level might not reflect active enzyme levels. Accordingly, dCK activity in lymphocytes should be assessed to better understand their differential sensitivity to 2CdA.

**Fig. 6:**
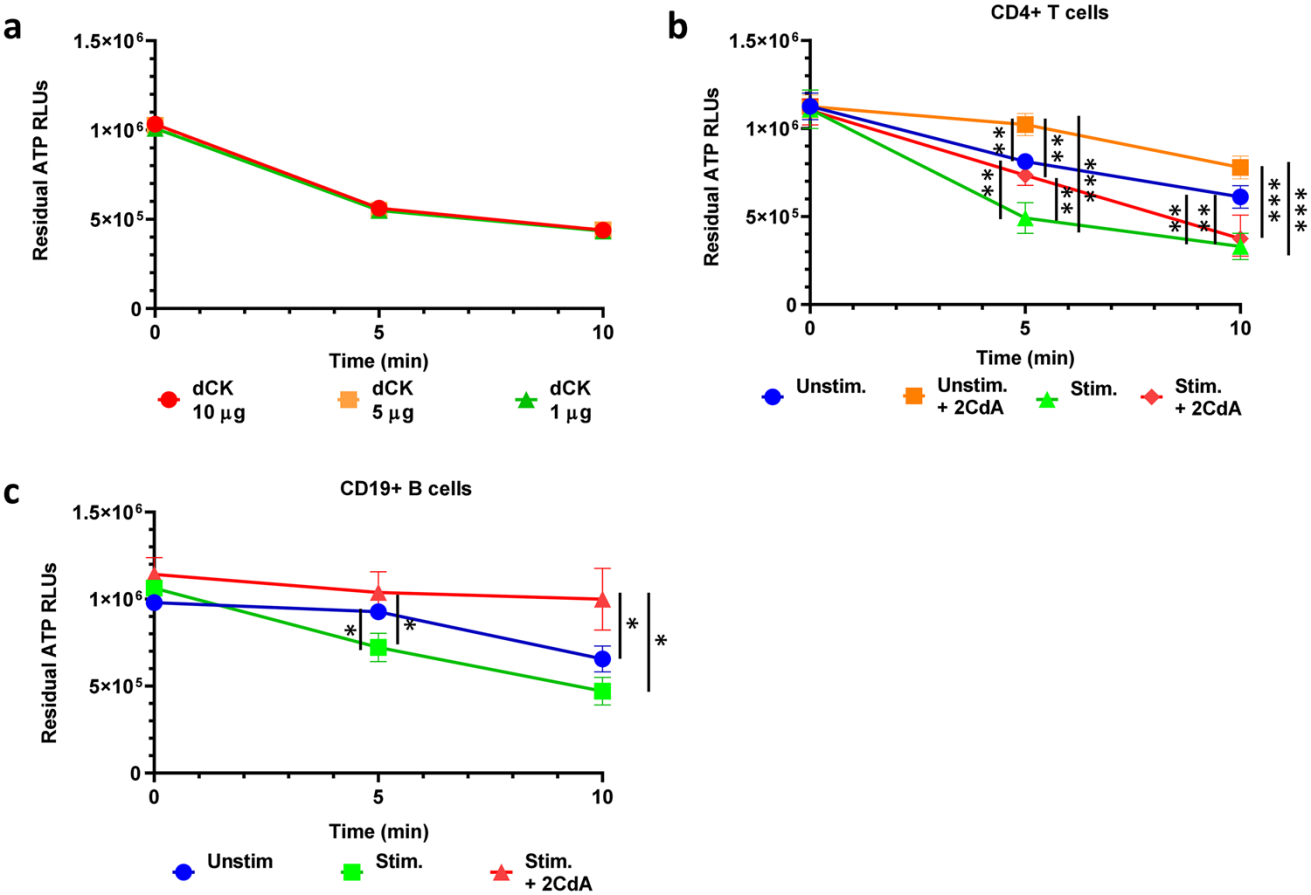
B- and T-cell activation increases dCK activity but exposure to 2CdA abrogates such activity in stimulated B cells. (**a**) Validation of dCK activity assay on different amounts of dCK in enriched preparation from PBMC. dCK activity is presented as RLUs), corresponding to the residual ATP from kinase reaction. Data are presented for PBMC isolated from 3 healthy donors assayed in independent experiments; (**b**) dCK activity in CD4 + T cells stimulated with anti-CD3/CD28 beads for 72 h in the presence or absence of 2CdA (500 nM). Data are presented for T cells isolated from 6 healthy donors assayed in independent experiments. *Unstim.* = unstimulated CD4 + T cells; *Unstim. + 2CdA* = unstimulated CD4 + T cells treated with 2CdA (500 nM); *Stim.* = CD4 + T cells stimulated with anti-CD3/CD28 beads; *Stim. + 2CdA* = CD4 + T cells stimulated with anti-CD3/CD28 beads and treated 2CdA (500 nM). (**c**) dCK enzymatic activity in CD19 + B cell stimulated with anti-IL-15/CpG for 72 h in the presence or absence of 2CdA (500 nM). Data are presented for B cells isolated from 6 healthy donors assayed in independent experiments. *Unstim.* = unstimulated CD19 + B cells; *Unstim. + 2CdA* = unstimulated CD19 + B cells treated with 2CdA (500 nM); *Stim.* = CD19 + B cells activated with IL-15/CpG; *Stim.+ 2CdA* = CD19 + B cells activated with IL-15/CpG treated with 2CdA (500 nM). * *P* < 0.05, ** *P* < 0.01, **** *P* < 0.0001

To be able to assess the enzymatic activity of dCK in cells, we have developed an in-house assay based on the enrichment of dCK from cell lysate through anion exchange chromatography (Hao et al. 2014) combined with a commercially available luminescence release assay (Fig. 1) that reflects residual ATP concentration in the reaction medium (expressed as RLUs), and thereby dCK enzymatic activity. The assay was developed and validated on different amounts of dCK (1, 5, and 10 µg) enriched from whole PBMC. As can be seen on Fig. 6a, there was an increase in ATP consumption (decrease in RLUs) reflecting increased enzymatic activity with time; as there was no dose-related difference in enzymatic activity, we thereafter selected 1 µg as an appropriate amount of enriched enzyme fraction to assess dCK activity under different experimental conditions.

We have applied this new assay to evaluate dCK activity upon stimulation of CD4 + T cells and the effect of 2CdA. As can be seen in Fig. 6b, T-cell stimulation led to a significant increase in dCK activity after a 5-minute incubation compared to unstimulated CD4 + T cells in the absence or presence of 2CdA, or to stimulated CD4 + T cells exposed to 2CdA; this difference disappeared after a 10-minute incubation in stimulated T cells exposed to 2CdA compared to non-exposed cells. The increase in dCK activity was maintained after a 10-minute incubation in stimulated compared to unstimulated CD4 + T cells in the absence or presence of 2CdA (Fig. 6b). dCK actvity in unstimulated CD4 + T cells exposed to 2CdA was significantly lower compared to the other experimental conditions after either a 5- or a 10-minute incubation (Fig. 6b).

CD19 + B cells activated with IL-15/CpG also displayed a higher dCK activity compared to unstimulated CD19 + B cells after 5- and 10-minute incubations (Fig. 6c). Upon exposure to 2CdA, dCK activity in IL-15/CpG-stimulated B cells was significantly lower than in untreated CD19 + B cells activated or not, after both 5 and 10 min of incubation.

## Discussion

The impact of 2CdA on mature lymphoid cell subpopulations has been extensively described in different disease contexts including MS (Baker et al. 2017; Comi et al. 2019; Garnache Ottou et al. 2014; Maitre et al. 2019; Sigal et al. 2010; Stuve et al. 2019). Clinical trials with 2CdA in MS have shown a sustained decrease in lymphocyte counts (Bell Gorrod et al. 2020). A moderate, but stable, depletion of CD4 + T cells was observed in MS patients treated with Mavenclad® tablets four or six months after the first treatment course, with a nadir of 50 % reduction from baseline (Stuve et al. 2019). A progressive recovery of CD4 + T cells was observed two months after the second treatment course (given 12 months after the first treatment course), starting from a 20 % nadir from baseline, with an approximate 45 % increase in cell numbers after 4 years (Comi et al. 2019). In contrast, 2CdA induced a marked depletion of CD19 + cells, up to 90 % at two months after the first treatment, which was followed by the rapid reconstitution of the B-cell population reaching 60 % baseline levels eight months after treatment. After the second treatment course, the B-cell population completely recovered from a 15 % nadir from baseline to baseline levels after two years (Comi et al. 2019). The difference in timing of immune reconstitution in B and T cells could result from differential susceptibility to 2CdA due to differences in dCK and NT5C2 expression and/or activity, depending on the cell activation status, in mature cells and/or in precursor cells within the bone marrow. Studies on the effects of 2CdA on hematopoietic precursors have shown that CD34 + CD38- cells are less susceptible to 2CdA-induced cell death than the more mature CD34 + CD38 + cells (Chow et al. 2002). The mechanisms underlying this differential susceptibility are yet unknown. In our study, no differences in dCK and NT5C2 protein expression and dCK/NT5C2 ratio were observed between CD34 + CD38- cells and CD34 + CD38 + cells (data not shown), nor in CLP and CMP. Previous studies used radiolabelled 5-D-[^3^H]deoxycytidine to show that immature lymphoid cells, particularly immature B cells isolated from human tonsils (of 3- to 6-year-old children) had a higher dCK activity than mature B cells (Staub 2006; Taljanidisz et al. 1987). These observations and our results on bone marrow progenitor cells might suggest that a higher dCK activity, rather than a higher amount of dCK protein, confers a preferential resistance to 2CdA in immature lymphoid cells compared to more mature subsets. Unfortunately, as the cell frequency of the immature lymphoid cell subsets in the bone marrow is low (10^5^ cells in 10^8^ bone marrow mononuclear cells) and our newly developed assay necessitates a relatively high cell number (around 10^6^ cells), we could not analyse dCK activity in these cell subsets.

In different mature lymphoid cell populations, the differential susceptibility to 2CdA could be related to specific dCK and NT5C2 expression patterns. However, we did not observe any significant differences in protein expression of dCK and NT5C2 or in dCK/NT5C2 ratio between Teff, Treg, B, or NK cells, or between subsets within each of these mature immune cell populations. Similarly, a study on B, T, and NK cells based on the data from a transcript microarray database (http://www.biogps.org; (Ceronie et al. 2018; Leist and Weissert 2011) also found that there were no significant differences in *DCK* and *NT5C2* mRNA expression between effector and memory T cells and between CD56low and CD56high NK cells. This same study revealed that memory B cells exhibit high levels of *DCK* mRNA, whereas immature B cells have lower levels (Ceronie et al. 2018). Accordingly, the authors suggested that the marked depletion of memory B cells eight months after 2CdA treatment could be explained by their high levels of the enzyme, whereas their lower levels of *DCK* would render immature B cells less sensitive to the drug (Ceronie et al. 2018). However, associating 2CdA sensitivity to a higher *DCK* mRNA expression in mature B cells compared to other B-cell subsets or other lymphoid cell subsets might only be part of a more complex picture. In our study, we found that cell activation was associated with an increase in dCK activity in both CD4 + T and CD19 + B cells. This is coherent with what was previously observed in germinal centers like mesenteric lymph nodes (Taljanidisz et al. 1987), tonsils, and other secondary lymphoid organs (Horváth et al. 1989), where dCK activity is increased in highly active lymphocytes. This is probably linked to the ability of this enzyme to supply cells with nucleotide precursors such as deoxycytidine, deoxyadenosine, deoxyguanosine and deoxythymidine (Staub 2006). Such functionality is particularly important in lymphocytes and other cell types (i.e. erythrocytes and neural cells) that are unable to synthetize nucleotides de novo and where salvage pathways, involving dCK and other enzymes, are used to recover bases and nucleosides that are formed during degradation of RNA and DNA (Austin et al. 2012; Toy et al. 2010). This suggests that highly active lymphocytes are more susceptible to 2CdA treatment because of their increased dCK activity as compared to lymphocytes in steady state, which results in increased accumulation of 2CdATP. In particular, we were able to confirm this hypothesis, as the apoptotic effect of 2CdA, previously observed in vitro only in PBMC (Korsen et al. 2015), was observed in stimulated, but not in unstimulated, CD4 + T cells. Altogether, these data, combined with the enhanced dCK activity we observed in stimulated T cells, could suggest that dCK activity, rather than expression, confers differential sensitivity to 2CdA in different lymphoid cell subsets. It could be further suggested that in the MS environment 2CdA is therefore more effective on active and proliferating lymphocytes, where an increased dCK activity is expected, hence affecting preferentially the potentially pathogenic Teff cells. However, no significant reduction in cell counts was observed between Teff cells and other T-cell subpopulations in MS patients after 2CdA treatment (Stuve et al. 2019). We observed that 2CdA has a similar impact on both unstimulated and stimulated CD19 + B cells with no significant difference in live cell counts under either condition. Interestingly in this context, it has been suggested on the basis of *DCK* mRNA expression data from microarray databases (Ceronie et al. 2018) that B cells are more susceptible to 2CdA than the other lymphoid cells. However, no difference in dCK expression was observed in our study between B cells and the other immune cell subsets analysed. Quantifying *DCK* mRNA and protein expression and, more importantly, activity in lymphoid cells from MS patients undergoing 2CdA treatment will be crucial to identify T- and B-cell subsets potentially relevant to MS pathogenesis that are preferential targets for this drug.

Very few studies have assessed the influence of 2CdA on dCK and NT5C2 expression in lymphocytes. In the present study, we observed for the first time that 2CdA treatment was associated with an overexpression of *DCK* mRNA in both stimulated CD19 + B and CD4 + T cells, results that were confirmed at protein level in CD4 + T cells. It could be suggested that the DNA damage caused by the accumulation of 2CdATP within the cell might trigger a failed attempt to produce new nucleotide precursors for DNA reparation, causing dCK overexpression. This theory has been already proposed to explain the increase in dCK activity in lymphocytes treated in vitro with 2CdA (Sasvari-Szekely et al. 1994; Staub 2006). Moreover, in stimulated CD4 + T cells exposed to 2CdA we observed an upregulation in *NT5C2* mRNA expression possibly suggesting a compensatory effect to mitigate the consequences of dCK overexpression. These results contrast with those from a previous study whereby 2CdA did not affect dCK protein expression in human tonsillar lymphocytes (Keszler et al. 2004). Discrepancies between our and that study could be related to the use of unstimulated lymphocytes together with their short 2CdA exposure time (i.e. 2 h) (Keszler et al. 2004).

We observed that 2CdA treatment of unstimulated CD4 + T cells was associated with a significant reduction in dCK activity compared to both stimulated CD4 + T cells exposed or not to 2CdA and unstimulated CD4 + T cells. In B cells, 2CdA was associated with a significant reduction in dCK activity in stimulated cells compared to stimulated or unstimulated cells that had not been exposed to 2CdA. This is the first time that a reduction in dCK activity is observed in lymphocytes after exposure to 2CdA; indeed, previous studies reported that the activity of dCK increased two to four times in human lymphocytes upon exposure to 2CdA, albeit after short-term incubations (i.e. 1-2 h) (Keszler et al. 2004; Sasvari-Szekely et al. 1994; Staub 2006). It could be speculated that the longer exposure to 2CdA, besides killing the majority of B cells that have higher dCK expression and/or activity, could select a B-cell population that has lower levels of the active enzyme. The presence of one or more such 2CdA-resistant B-cell subset(s), potentially belonging to immature B cells as suggested (Ceronie et al. 2018), could explain the progressive B-cell reconstitution following cell depletion that has been observed in clinical trials (Comi et al. 2019; Stuve et al. 2019). 2CdA exposure, in contrast, did not affect dCK activity in stimulated T cells. This could possibly be due to a compensatory effect of anti-CD3/CD28 stimulation, which we showed to be associated to an increase in dCK expression and activity.

Altogether, the results of this work highlight the importance of measuring dCK activity to better dissect the impact of 2CdA treatment on B and T cells according to their activation status. Thus, 2CdA might enhance its effect on B and T cells by upregulating dCK and NT5C2 expression and by influencing dCK activity. The possibility to assay dCK activity in cells, as demonstrated in this in-vitro study, will also be crucial to further understand how 2CdA treatment might affect dCK activity in lymphocytes from MS patients. Lymphocyte reduction is part of the mechanism of action of 2CdA, but long-term efficacy is seen beyond lymphocyte recovery. In order to better understand the immunological mechanisms driving long-term efficacy, it would be of interest to investigate the different susceptibilities of B- and T-cell subsets in patients showing either long-term treatment response or disease reactivation. In this context, we have now started a longitudinal study in MS patients undergoing treatment with Mavenclad tablets to assess whether measurement of dCK kinase activity in patients with MS could serve as a potential biomarker of lymphocyte response to 2CdA.

## Supplementary Information

Below is the link to the electronic supplementary material.

Supplementary Material 1 Figure 1. Gating strategy to define viable CMP and CLP subsets in BM mononuclear cells and their dCK and NT5C2 expression (a) The procedure involves first the evaluation of BM mononuclear cell viability using FVS-780 marker and the definition of these cells by their morphology. (b) Lineage mAb Cocktail (CD3, CD14, CD16, CD19, CD20, CD56) was used to separate lineage-negative immature cell populations from mature cell subsets. (c) Gating to distinguish CD38+CD34+ mature cell progenitors from CD38+CD34- stem cells. (d) CMP and (e) CLP are defined according to their expression of CD123 and CD45, or of CD10, respectively. (f) dCK and NT5C2 expression, shown as MFI, in CMP and CLP respectively.

Supplementary Material 2 Figure 2. Gating strategy to define B-, NK- and T-cell subsets (a) Boolean gating strategy is used to define T effector subsets as Th1 = Q1 and CD161- ; Th17 = Q4, CD161+ and CCR4+ ; Th17/1 = Q2, CD161+ and CCR4- . (b) Gating strategy for Treg cell subsets. (c) Gating strategy for B and NK cells.

## Data Availability

All data generated or analysed during this study are included in this published article and its supplementary information files.
