# Supplementary figures and images for: Different Susceptibility of T and B Cells to Cladribine Depends On Their Levels of Deoxycytidine Kinase Activity Linked to Activation Status
Source: J Neuroimmune Pharmacol. 2021 Apr 14;17(1-2):195–205. doi: 10.1007/s11481-021-09994-3 (PMC9726780; doi:10.1007/s11481-021-09994-3)

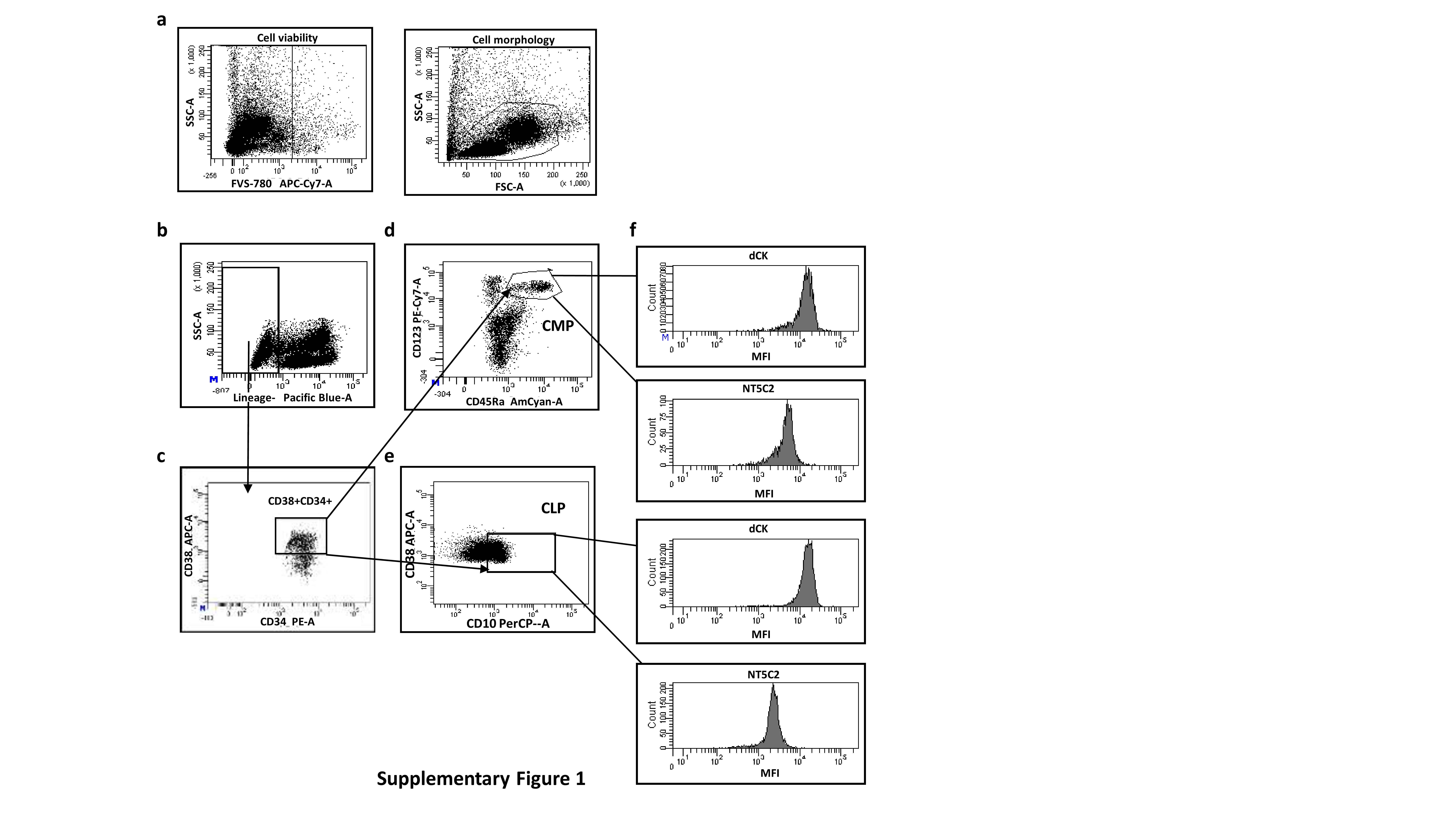

Supplement: Supplementary file 1 — Supplementary Material 1 Figure 1. Gating strategy to define viable CMP and CLP subsets in BM mononuclear cells and their dCK and NT5C2 expression (a) The procedure involves first the evaluation of BM mononuclear cell viability using FVS-780 marker and the definition of these cells by their morphology. (b) Lineage mAb Cocktail (CD3, CD14, CD16, CD19, CD20, CD56) was used to separate lineage-negative immature cell populations from mature cell subsets. (c) Gating to distinguish CD38+CD34+ mature cell progenitors from CD38+CD34- stem cells. (d) CMP and (e) CLP are defined according to their expression of CD123 and CD45, or of CD10, respectively. (f) dCK and NT5C2 expression, shown as MFI, in CMP and CLP respectively. [file 11481_2021_9994_MOESM1_ESM.tiff]

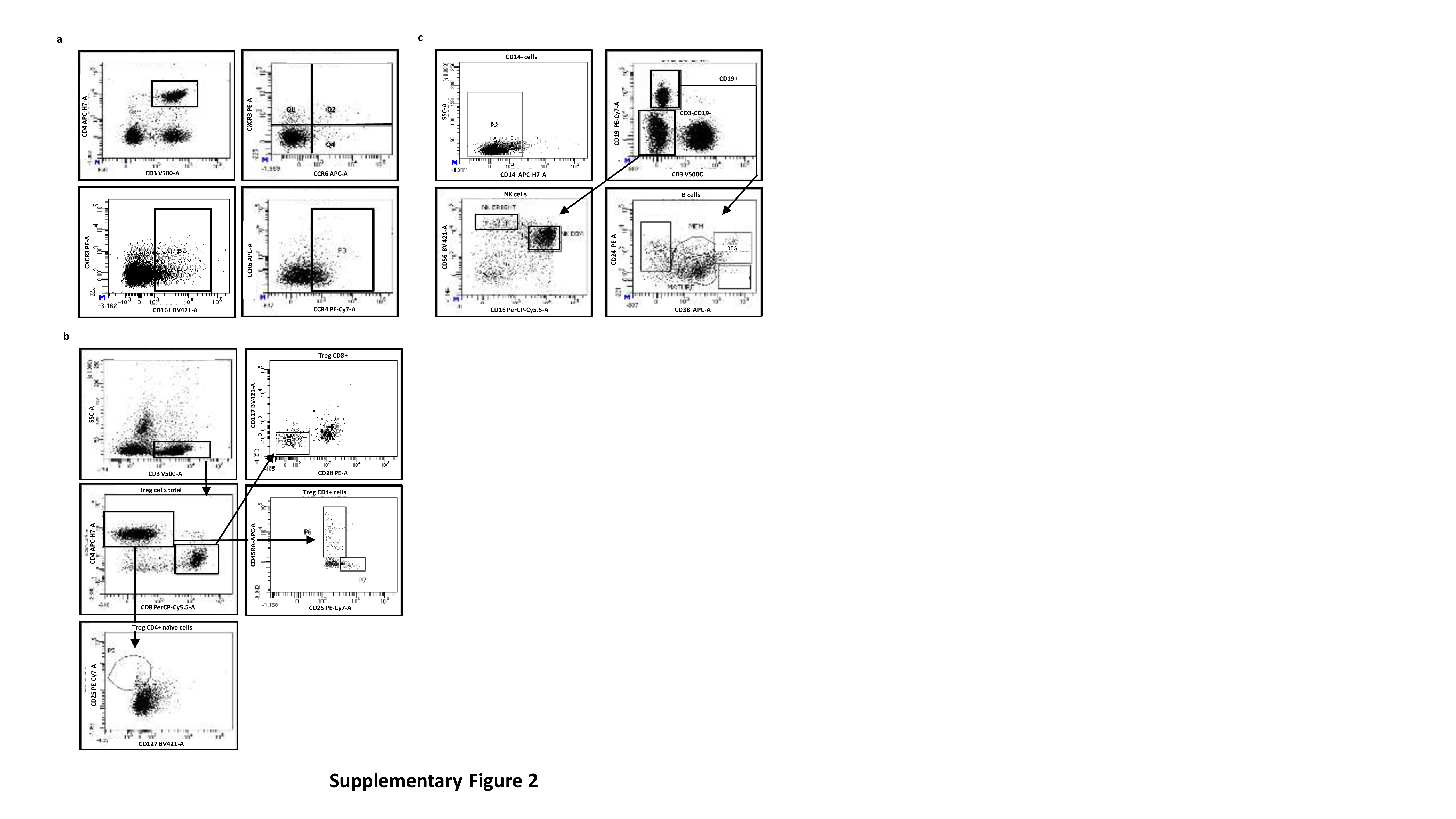

Supplement: Supplementary file 2 — Supplementary Material 2 Figure 2. Gating strategy to define B-, NK- and T-cell subsets (a) Boolean gating strategy is used to define T effector subsets as Th1 = Q1 and CD161- ; Th17 = Q4, CD161+ and CCR4+ ; Th17/1 = Q2, CD161+ and CCR4- . (b) Gating strategy for Treg cell subsets. (c) Gating strategy for B and NK cells. [file 11481_2021_9994_MOESM2_ESM.tiff]
